# Genetic Structuration, Demography and Evolutionary History of *Mycobacterium tuberculosis* LAM9 Sublineage in the Americas as Two Distinct Subpopulations Revealed by Bayesian Analyses

**DOI:** 10.1371/journal.pone.0140911

**Published:** 2015-10-30

**Authors:** Yann Reynaud, Julie Millet, Nalin Rastogi

**Affiliations:** WHO Supranational TB Reference Laboratory, Tuberculosis and Mycobacteria Unit, Institut Pasteur de la Guadeloupe, Abymes, Guadeloupe, France; University of Minnesota, UNITED STATES

## Abstract

Tuberculosis (TB) remains broadly present in the Americas despite intense global efforts for its control and elimination. Starting from a large dataset comprising spoligotyping (n = 21183 isolates) and 12-loci MIRU-VNTRs data (n = 4022 isolates) from a total of 31 countries of the Americas (data extracted from the SITVIT2 database), this study aimed to get an overview of lineages circulating in the Americas. A total of 17119 (80.8%) strains belonged to the Euro-American lineage 4, among which the most predominant genotypic family belonged to the Latin American and Mediterranean (LAM) lineage (n = 6386, 30.1% of strains). By combining classical phylogenetic analyses and Bayesian approaches, this study revealed for the first time a clear genetic structuration of LAM9 sublineage into two subpopulations named LAM9C1 and LAM9C2, with distinct genetic characteristics. LAM9C1 was predominant in Chile, Colombia and USA, while LAM9C2 was predominant in Brazil, Dominican Republic, Guadeloupe and French Guiana. Globally, LAM9C2 was characterized by higher allelic richness as compared to LAM9C1 isolates. Moreover, LAM9C2 sublineage appeared to expand close to twenty times more than LAM9C1 and showed older traces of expansion. Interestingly, a significant proportion of LAM9C2 isolates presented typical signature of ancestral LAM-RD^Rio^ MIRU-VNTR type (224226153321). Further studies based on Whole Genome Sequencing of LAM strains will provide the needed resolution to decipher the biogeographical structure and evolutionary history of this successful family.

## Introduction

With an estimated 9 million new cases (range: 8.6–9.4 million) and 1.5 million deaths yearly (range: 1.3–1.7 million), tuberculosis (TB) remains a major public health problem globally [1]. Integrated strategies for controlling the disease need to be implemented based on efficient diagnostics targeting recent transmission chains and outbreaks leading to adapted tailored therapy. In such a context, knowing with great resolution the epidemiology at different spatial and temporal scales is of prime importance for local and global TB control and a *sine qua non* condition for detection of fluctuations in TB population dynamics. Indeed, *Mycobacterium tuberculosis* complex genotypic lineages have emerged during past several thousand years due to co-adaptation with its human host, and the intricate relationship it maintains with its host is largely responsible for its proven phylogeographical specificities.

Molecular genetic studies of circulating *M*. *tuberculosis* strains using various genotyping technologies allow to monitor strain dispersal and evolutionary adaptations, important to stem bacterial and disease spread. These include classical genotyping tools such as IS*6110*-RFLP [2], CRISPRs (Clustered Regularly Interspaced Short Palindromic Repeats)–based spoligotyping [3], MIRU-VNTRs (Mycobacterial Interspersed Repetitive Unit—Variable Number of Tandem Repeats) [4], and RD-LSPs (Regions of Differences—Large Sequence Polymorphisms) [5], which defined six major lineages: Indo-Oceanic (lineage 1), East-Asian including Beijing (lineage 2), East-African-Indian (lineage 3), Euro-American (lineage 4), West Africa or *M*. *africanum* I (lineage 5), and West Africa or *M*. *africanum* II (lineage 6). Furthermore, a new lineage referred to as lineage 7 was recently described in Ethiopia and the Horn of Africa [6]. More recently, based on Whole Genome Sequencing (WGS), a robust SNP (Single Nucleotide Polymorphism) barcode was developed to infer phylogenetic relationships both inter- and intra-lineage to an unprecedented level of resolution [7].

The aim of the present study was to get an overview of strains circulating in the Americas where TB remains broadly present despite intense global efforts for its control and elimination. The *M*. *tuberculosis* strains currently circulating in Americas were brought by Europeans with the Euro-American lineage 4 being the most predominant [8], highlighting past and present European colonial and cultural influence on the current TB situation [9,10]. Among the large and heterogeneous lineage 4, the Latin American Mediterranean (LAM) family was first suggested based on the phylogenetic analysis of a large spoligotyping dataset and its name reflects the strains’ origin [11]. LAM lineage comprising several sublineages is the largest and most widespread within the Euro-American lineage 4; the phylogenetical inclusion of some sublineages within this group has been recently questioned [12,13]; however, only few studies have been conducted on genotypic structure and phylogenetic history of this efficient genogroup. Consequently, this study aimed to get a first detailed overview of LAM lineage genetic specificities circulating in the Americas, and further provides novel evidence regarding LAM9 genetic structuration as two subpopulations categorized by distinct evolutionary histories and demographic characteristics.

## Materials and Methods

### Data collection

This study made use of genotyping information of *M*. *tuberculosis* clinical isolates fully available without restriction. All data used in our study are either available at: http://www.pasteur-guadeloupe.fr:8081/SITVIT_ONLINE, or in published studies [8,9,13–15,30–55]. This database currently centralizes data on 120000 *M*. *tuberculosis* complex strains from 170 countries. Spoligotype International Type (SIT) and MIRU International Type (MIT) designates an identical pattern shared by two or more patient isolates by spoligotyping and MIRU-VNTRs, whereas “orphan” designates patterns reported for a single isolate not reported before in the database. Phylogenetic clade assignation follows rules of SITVITWEB in which the LSP-based Euro-American lineage (lineage 4) [5] is split in LAM, ill-defined T, Haarlem (H), X, and S lineages; the LSP-based “indo-Oceanic” lineage is named East-African Indian (EAI) by spoligotyping, while EAI by LSPs corresponds to Central-Asian (CAS) in the SITVIT2 database. Lineages were subdivided into sublineages as described recently [15]. For the purpose of this study, we exclusively focused on spoligotyping and 12-loci MIRU-VNTRs of *M*. *tuberculosis* isolated in the Americas in 31 countries.

### Phylogenetic inferences

BioNumerics software 6.6 (Applied Maths, Sint-Martens-Latem, Belgium) was used to compare spoligotypes and 12-loci MIRU-VNTR patterns of *M*. *tuberculosis* isolates from Americas, by drawing Minimum Spanning Trees (MSTs) in order to visualize evolutionary relationships between the clinical isolates in our study. MSTs are undirected graphs in which all samples are connected together with the fewest possible connections between nearest neighbors.

### Exploration of LAM9 sublineage population structure

After an initial analysis of lineage 4 *M*. *tuberculosis* isolates, the available evidence suggested a possible subdivision of LAM9 strains into two distinct subpopulations. To confirm this hypothesis, population structure of all LAM9 isolates with data available on 12-loci MIRU-VNTRs (n = 450) was inferred by using a Bayesian model approach implemented in the software STRUCTURE 2.3 [16]. An admixture model was implemented in 10 parallel Markov chains for K values ranging from 1 to 5, with a burn-in of 100000 iterations and a run length of 10^6^ iterations following the burn-in. This admixture model can deal with complexities of real data and considers that individuals may have mixed ancestry and may have inherited part of their genome from ancestors in population *k*. To estimate the number of population among LAM9 isolates, delta K was calculated by the Evanno method [17] implemented in the program STRUCTURE HARVESTER [18]. Medians were then calculated from 10 replicates for K = 2 by using the FullSearch algorithm implemented in CLUMPP 1.1.2 software [19] to guarantee the optimum clustering. A cutoff of 0.75 was fixed for clustering of LAM9 isolates to subpopulation 1 or 2. Finally, estimated membership coefficients were visualized using the software DISTRUCT 1.1 [20]. A new MST analysis was then performed using BioNumerics software 6.6 and identifying LAM9 strains belonging to subpopulation 1 or 2 defined by STRUCTURE analysis.

### Allelic richness

For analyses on allelic richness, *M*. *tuberculosis* strains were grouped according to their clades defined by the MST analysis, followed by structuration of LAM9 subpopulations as defined by STRUCTURE software. Mean allelic richness of each *M*. *tuberculosis* lineages was evaluated where 12-loci MIRU-VNTRs were available for at least 25 samples using the software HP-RARE 1.0 [21]. This approach uses the statistical technique of rarefaction which compensates for sampling disparity.

### LAM9 coalescence and demography

To explore the most probable past demographic changes, a Bayesian based coalescent approach [22,23] implemented in the Msvar 1.3 algorithm was applied on LAM9 sublineage strains (n = 450) using 12-loci MIRU-VNTR data. The loci are assumed to be evolving by a stepwise mutation model (SMM) [24–26]. Posterior distribution of demographic and genealogical parameters were inferred by Markov Chain Monte Carlo (MCMC) simulations. The assumed demographic history is of a past population of size N1 that experienced a change in size at some time t_a_ in the past to reach current effective population size N0. We tested hypothesis of declining population (10^−2^ and 10^−3^ as a prior) where expansion ratio R = N0/N1<1, of stable population where R = 1 and of expanding populations (10^1^ to 10^3^ as a prior) where R>1. The analyses were performed assuming exponential demographic change. The prior mutation rate value of each MIRU-VNTR locus ranged between 10^−8^ and 10^−9^ per locus and per generation, according to previous studies [25,27,28]. The chain was run for 2 billion steps, recording parameter values every 100000 steps. The MCMC output was analyzed using the software Tracer [29] to obtain the posterior distribution and the effective sample size (ESS) of all parameters (which were all above 140) after a burn-in of 10%.

### Ethics statements

None required since the genotyping data extracted from the SITVIT2 database was anonymized.

## Results and Discussion

### Distribution of *M*. *tuberculosis* lineages in the Americas

Majority of data regarding distribution of *M*. *tuberculosis* lineages and sublineages in the Americas has been published in earlier individual studies focusing on respective population structures within a country [8,9,13–15,30–55]. However, to have a global overview of mapping at the level of the continent, we hereby analyze metadata allowing greater resolution in order to more deeply explore the genetic structuration of predominant lineages on a total of 21183 *M*. *tuberculosis* isolates from America (31 countries). Spoligotype profiles were available for all the 21183 strains studied, while 12-loci MIRU-VNTR profiles were available for a total of 4022 isolates.

Starting from 21183 *M*. *tuberculosis* isolates, a total of 17119 (80.8%) strains belonged to lineage 4 (Euro-American) according to spoligotyping [56] (Table 1). This widely predominant lineage supports the hypothesis of a European dissemination from either early settlement or trade associations [8–10]. Among the lineage 4 strains, a total of 6386 (30.1%) belonged to the LAM lineage; 4843 (22.9%) belonged to the T lineage; 3699 (17.5%) belonged to the H (Haarlem) lineage; 1564 (7.4%) belonged to the X lineage; 1163 belonged to the EAI lineage; 1105 belonged to the Beijing lineage; 497 (2.3%) belonged to the S lineage; 162 (0.8%) belonged to the CAS lineage; 102 (0.5%) belonged to the MANU lineage; 94 (0.4%) belonged to the AFRI lineage; 67 (0.3%) belonged to the Cameroon lineage and 63 (0.3%) belonged to the URAL lineage. Among predominant lineages LAM, T and H, the main sublineages were respectively: LAM9 (33.7%), T1 (72.3%), and H3 (65.5%). LAM9 alone represented 10.15% of all *M*. *tuberculosis* strains (n = 21183) from the Americas (for distribution of predominant SITs in the global database, readers may refer to S1 Table).

**Table 1:** Distribution of main *M*. *tuberculosis* lineages and sublineages in Americas according to SITVIT2 database (n = 21183 strains) based on spoligotyping.

Lineages	N	% vs total	Sublineage	N	% intra lineage
LAM	6386	30,1	LAM9	2151	33,7
			LAM3	1034	16,2
			LAM2	762	11,9
			LAM1	611	9,6
			LAM6	549	8,6
			LAM5	507	7,9
			LAM NT	392	6,1
			LAM4	316	5,0
			LAM11-ZWE	29	0,5
			LAM8	27	0,4
			LAM12-Madrid1	8	0,1
T	4843	22,9	T1	3499	72,3
			T2	434	9,0
			T3	246	5,1
			T NT	223	4,6
			T4-CEU1	110	2,3
			T5	89	1,8
			T5-Madrid2	89	1,8
			T4	52	1,1
			T-H37Rv	29	0,6
			T5-RUS1	27	0,6
			T2-uganda	17	0,4
			T3-ETH	10	0,2
			T1-RUS2	7	0,1
			T-tuscany	7	0,1
			T3-OSA	4	0,1
H	3699	17,5	H3	2422	65,5
			H1	993	26,9
			H2	270	7,3
			H NT	14	0,4
X	1564	7,4	X3	589	37,7
			X1	534	34,1
			X2	434	27,8
			X NT	7	0,5
EAI	1163	5,5	EAI2-Manila	406	34,9
			EAI5	336	28,9
			EAI6-BGD1	132	11,4
			EAI1-SOM	112	9,6
			EAI3-IND	100	8,6
			EAI4-VNM	31	2,7
			EAI2-nonthaburi	23	2,0
			EAI2	9	0,8
			EAI7-BGD2	7	0,6
			EAI8-MDG	5	0,4
			EAI NT	2	0,2
Beijing	1105	5,2	-	-	-
S	497	2,3	-	-	-
CAS	162	0,8	CAS1-Delhi	95	58,6
			CAS NT	49	30,3
			CAS1-Kili	11	6,8
			CAS2	7	4,3
MANU	102	0,5	MANU2	55	53,9
			MANU1	32	31,4
			MANU3	12	11,8
			Manu_ancestor	3	2,9
AFRI	94	0,4	AFRI_2	42	44,7
			AFRI_1	30	31,9
			AFRI NT	16	17,0
			AFRI_3	6	6,4
Cameroon	67	0,3	-	-	-
URAL	63	0,3	Ural-1	52	82,5
			Ural-2	11	17,5
Unknown	1438	6,8	-	-	-

**Fig 1:**
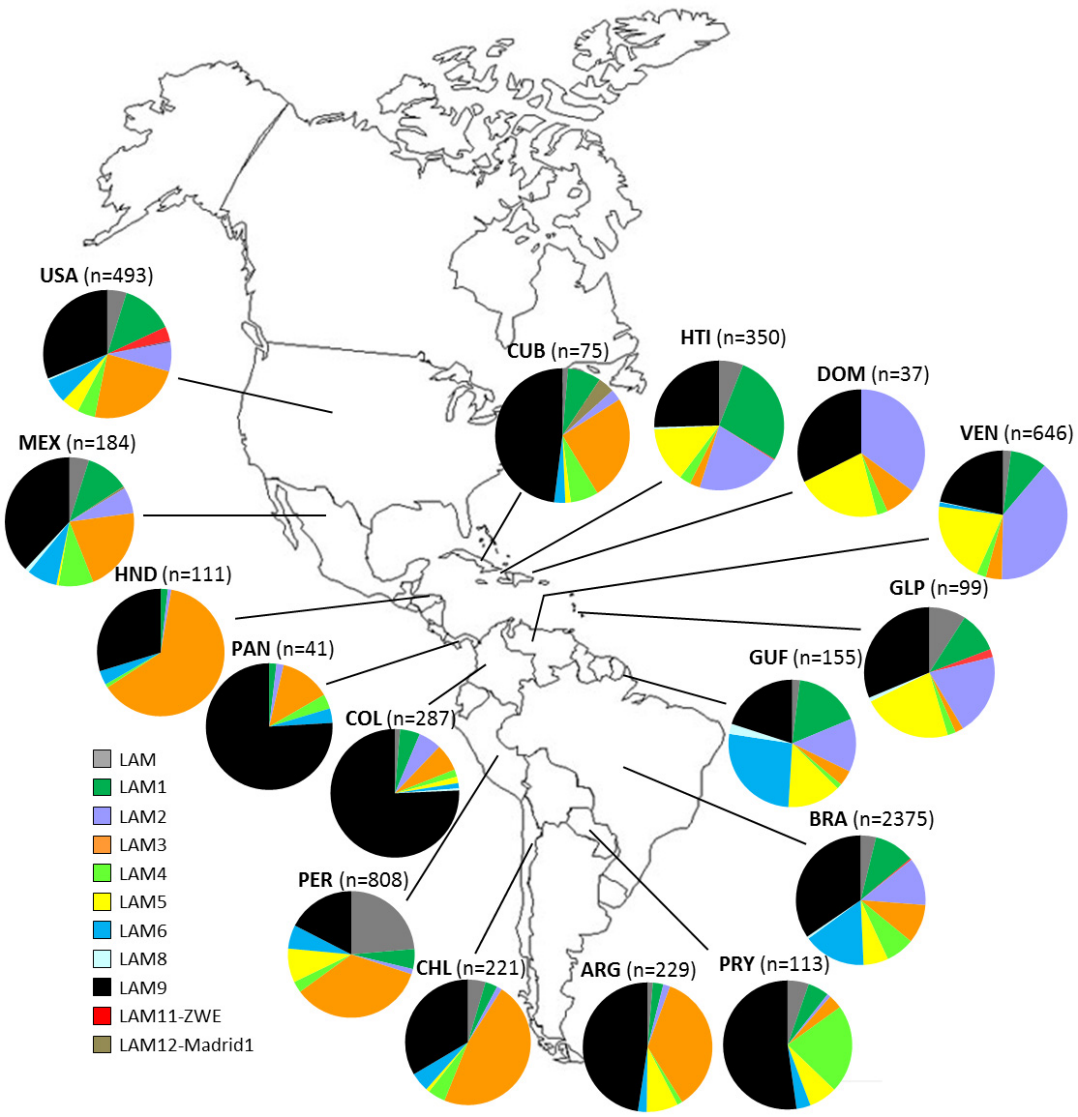
Geographic distribution of LAM sublineages in various countries of Americas (when n>36). Phylogenetic clade assignation using spoligotyping follows rules of SITVITWEB database. Country codes are shown as ISO 3166–1 alpha-3 code.

When focusing on geographical distribution of LAM sublineages in the Americas (Fig 1), contrasted patterns of sublineages proportions were observed; briefly: (i) Guadeloupe, Venezuela and Haiti presented quite similar distribution patterns with predominance of LAM9, LAM2, LAM5 and LAM1 sublineages, and two related patterns in respectively, (ii) Dominican Republic with absence of LAM1 and (iii) in French Guiana and Brazil with presence of LAM6; four other distribution patterns were characterized by (iv) large predominance of LAM9 isolates in Panama and Colombia, (v) predominance of LAM9 and LAM3 in USA, Cuba, Mexico, Peru, Chile and Argentina, (vi) large predominance of LAM3 in Honduras, and (vii) predominance of LAM9 and LAM4 in Paraguay. Even if these differences could be caused by differences in sample size, the probable relationship to respective immigration history and demographic expansion of the strains initially introduced should be further explored. For having an overview of global distribution of all *M*. *tuberculosis* lineages in the Americas, readers may refer to S1 Fig, which is an updated version of a distribution map published recently [30], as well as distribution maps of two other predominant lineages T and H (S2 and S3 Figs).

### Genetic structuration of LAM9 sublineage

**Fig 2:**
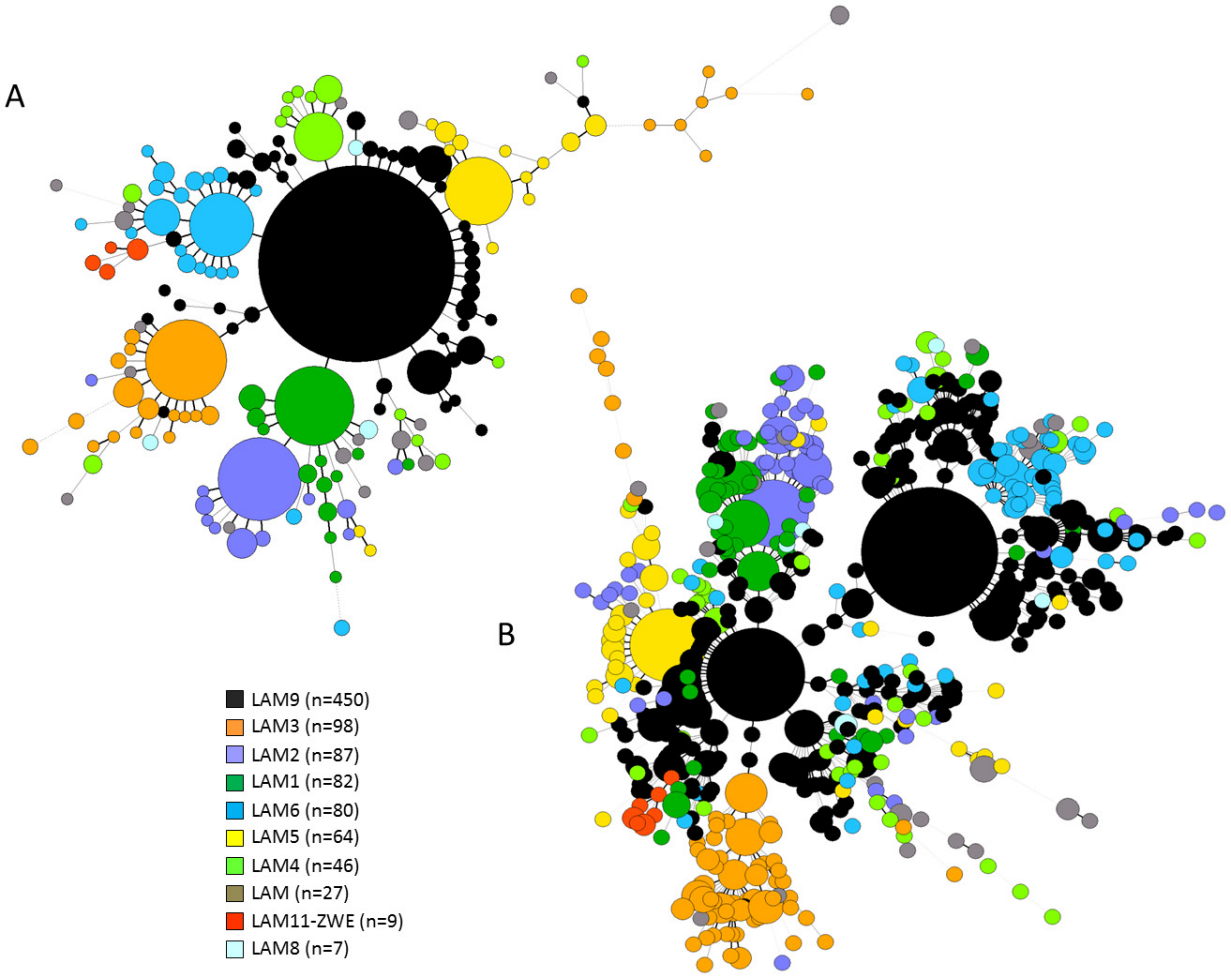
Minimum Spanning Tree (MST) illustrating evolutionary relationships between *M*. *tuberculosis* LAM lineage isolates (n = 950). The analysis is based on spoligotyping used alone (A), and combination of spoligotypes and 12-loci MIRU-VNTR markers (B). The MST connects each genotype based on degree of changes required to go from one allele to another; the complexity of the lines denotes the number of allele/spacer changes between two patterns: solid lines (1 or 2 or 3 changes), gray dashed lines (4 changes) and gray dotted lines (5 or more changes); the size of the circle is proportional to the total number of isolates sharing same pattern.

**Fig 3:**
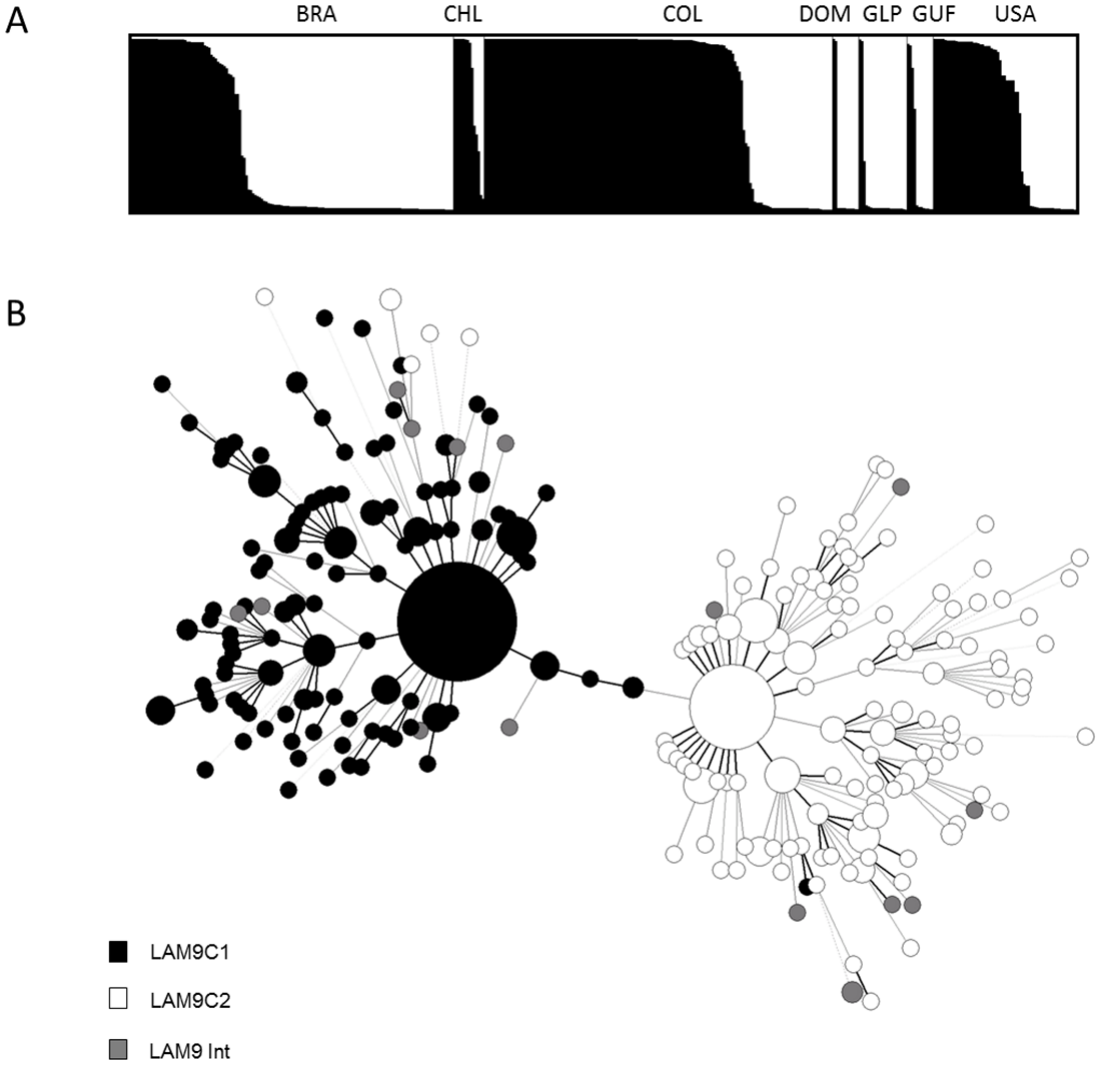
Evolutionary relationships of the LAM9 sublineage isolates (n = 450). (A) Geographical distribution and LAM9C1 and C2 isolates defined by Bayesian cluster analysis using STRUCTURE software run on 12-loci MIRU-VNTRs. Each of the strains is represented by a thin vertical line, partitioned into black or white segments that represent the strains estimated proportion of membership in clusters LAM9C1 and LAM9C2 respectively. (B) MST analysis on combined spoligotyping and MIRU-VNTR data for strains prelabeled as LAM9C1 (n = 226) and C2 (n = 208) based on previous STRUCTURE analysis (strains in intermediate position between C1 and C2 are indicated as LAM9 Int, n = 16). The complexity of the lines denotes the number of allele/spacer changes between two patterns while the size of the circle is proportional to the total number of isolates sharing same pattern. Country codes are shown as ISO 3166–1 alpha-3 code.

Evolutionary relationships between all LAM lineage isolates pooled together for which both the spoligotyping and 12-loci MIRU-VNTR data were available (n = 950) were investigated by MST analysis. Spoligotyping alone showed a closely-structured phylogenetic tree of this superfamily (Fig 2A), with a huge central node made-up of the LAM9 sublineage; an observation also confirmed on global spoligotyping data on all LAM9 strains (n = 2151 strains, data not shown). However, it is common knowledge that mainly because of homoplasy, spoligotyping has limited resolution power when inferring *M*. *tuberculosis* phylogeny and that discrepancies can be obtained when comparing spoligotyping and other genotyping approaches as for example MIRU-VNTRs, IS*6110* and LSPs [8,12,13,31,57,58]. It is therefore of prime importance to perform polyphasic analyses when exploring *M*. *tuberculosis* evolution for adequate discrimination. For this reason, we further looked in the genetic structuration of LAM lineage strains by constructing a MST based on combined spoligotyping and MIRU-VNTR data (Fig 2B), which globally conserved the overall structuration observed for all LAM sublineages with the exception of LAM9 (n = 450 strains in total) which was clearly split into two distinct subpopulations, an observation not yet reported in literature. To confirm this subdivision, a Bayesian model approach using STRUCTURE 2.3 software [16] was performed on same LAM9 dataset using 12-loci MIRU-VNTRs. The appropriate K value was selected by the Evanno method [17] (S4 Fig). STRUCTURE identified a total of K = 2 deeply divergent populations, named LAM9 clusters C1 and C2 (Fig 3A); individual strains in this figure are represented by vertical lines divided into two colored segments with the length of each segment being proportional to the estimated membership in each of the two populations (cutoff = 0.75). By this analysis, a total of 226 isolates belonged to LAM9C1 and 208 isolates belonged to LAM9C2. We further checked the congruence of these results by performing an additional MST analysis of strains prelabeled as LAM9C1 and C2 based on STRUCTURE analysis. The resulting phylogenetic tree (Fig 3B) showed congruent results between both approaches. Briefly, 99.6% (n = 225/226) of isolates defined as LAM9C1 by STRUCTURE analysis were conserved in the same group by MST analysis, as well as 97.1% (n = 202/208) of LAM9C2 isolates. Last but not least, the star-like structure observed for both LAM9 subpopulations in Fig 3B is compatible with their recent clonal expansion.

### Geographical distribution, demography and genetic characteristics of LAM9C1 and C2 subpopulations

When focusing on geographical distribution of LAM9C1 and C2 isolates (Fig 3A), it appears that LAM9C1 is predominant in Chile (64.3%, n = 9/14), Colombia (74.2%, n = 118/159) and USA (56.9%, n = 37/65), while LAM9C2 is predominant in Brazil (64.6%, n = 95/147), Dominican Republic (83.3%, n = 10/12), Guadeloupe (86.4%, n = 19/22) and French Guiana (66.7%, n = 8/12). These results suggest a phylogeographical specificity of these two subpopulations even if differences could be caused by differences in sample size. Allelic richness of 12-loci MIRU-VNTR markers was evaluated for LAM9C1 and C2 groups globally as well as at country level in Brazil, Colombia and USA, using a rarefaction procedure implemented in HP-RARE 1.0 software [21] (Table 2). Both globally as well as for each of the countries studied, LAM9C2 was characterized by higher allelic richness than LAM9C1 isolates. Taking allelic richness as a surrogate marker of diversification time, our results tend to suggest that LAM9C2 isolates are older than LAM9C1 ones. Furthermore, it is interesting to note that, allelic richness was smaller for both LAM9 populations in Colombia as compared to Brazil and USA, probably reflecting respective immigration histories—an observation also seen through the preponderance of LAM9 representing 75% of all *M*. *tuberculosis* strains in Colombia (Fig 1).

**Table 2:** Allelic richness ± standard deviation (SD) of LAM9C1 and C2 subpopulations according to country of isolation.

Sublineages and country of isolation	Mean allelic richness	±SD
LAM9C1	2,06	1
LAM9C2	2,25	0,9
LAM9C1 BRA	2,15	1,04
LAM9C2 BRA	2,4	0,91
LAM9C1 COL	1,62	0,7
LAM9C2 COL	1,83	1,08
LAM9C1 USA	2,04	0,96
LAM9C2 USA	2,21	0,97

Allelic richness is evaluated for 12-loci MIRU-VNTRs using a rarefaction procedure (when n>25 per lineage and per country); countries names are defined by ISO 3166–1 alpha-3 code.

**Fig 4:**
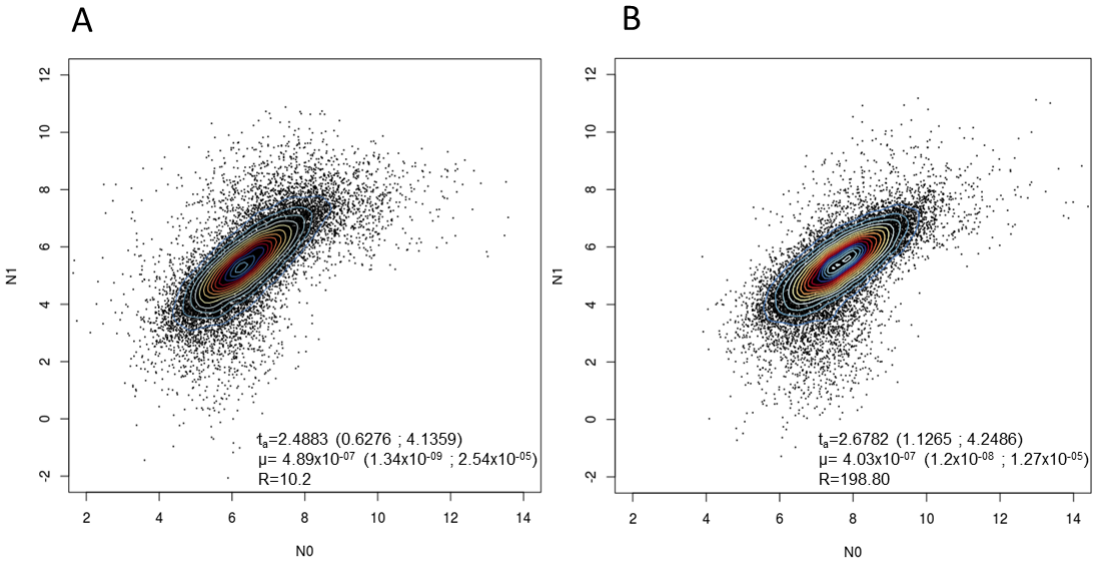
12-loci MIRU-VNTR based demographic and dating estimates of LAM9 sublineages inferred by a Bayesian approach on Msvar 1.3 algorithm. (A) 2D Kernel density plots producing a smooth estimate of the density of the marginal posterior distribution of N0 the current effective population size, and N1 the population size before expansion (in log scale) for LAM9C1 isolates. (B) Same figure for LAM9C2 isolates. t_a_, time elapsed since last expansion began expressed in years (log scale); R = N0/N1 traduce median value of expansion ratio; μ, mutation rate per locus and per generation. All estimates correspond to median values, followed by 95% highest posterior densities indicated in parentheses.

Recent demographic changes of LAM9C1 and LAM9C2 isolates were inferred from a Bayesian-based coalescent approach available for MIRU-VNTR markers and implemented in the Msvar 1.3 algorithm [22,23] (Fig 4). As prior we tested scenario for recent expansion, decrease of bacterial population size or stable population size. We then calculated the time t_a_ since last expansion and mutation rate μ per locus and per generation. Although both subpopulations were characterized by strong expansion, LAM9C2 expansion rate was twenty times higher than LAM9C1 (expansion ratio R of 198.8 vs. 10.2). Furthermore, VNTR based dating estimates suggested older traces of expansion dating to 480 years for LAM9C2 isolates vs. 300 years for LAM9C1 (Fig 4). Even if these results should be taken with caution considering large confidence intervals and uncertainties in mutation rates, these dating estimates were synchronous with immigration from the Old World to the New World. Further WGS based studies should help to better understand the past history of LAM sublineages in the Americas.

When focusing on MIRU-VNTR markers driving structuration of LAM9 isolates into two subpopulations (Table 3), a total of four markers clearly present contrasted number of repeats between sublineages: MIRU2, MIRU16, MIRU31 and MIRU40. Indeed, MIRU2 and MIRU40 were highly discriminatory. For MIRU2, 94.2% (n = 213/226) of LAM9C1 isolates presented a single repeat vs. 0.5% (n = 1/208) for LAM9C2 isolates, and 96.2% (n = 200/208) of LAM9C2 isolates presented a double repeat vs. 5.3% (n = 12/226) for LAM9C1 isolates. For MIRU40, 86.1% (n = 179/208) of LAM9C2 isolates showed a single repeat vs. 0.4% (n = 1/226) for LAM9C1. Interestingly, these same MIRU loci were shown to be highly discriminatory for LAM-RD^Rio^ vs. “wild type” (WT) LAM isolates [59]: 100% of LAM-RD^Rio^ and just 2% of WT LAM patient strains had a single copy at MIRU40 while 98% of LAM-RD^Rio^ had two copies at MIRU2. Indeed, the authors of this study proposed to combine these markers to identify RD^Rio^ strains within databases listing MIRU-VNTR typed LAM strains and more specifically to identify the theoretical “founding MIRU-VNTR type” for RD^Rio^ *M*. *tuberculosis* (224226153321). Because LAM9C2 isolates in our study present typical signature of LAM-RD^Rio^ strains, one may hypothesize that LAM9C2 could be constituted by significant number of LAM-RD^Rio^ isolates, and more precisely by 27.9% (n = 58/208) of the hypothetical ancestral RD^Rio^ MIRU-VNTR type (224226153321). This observation merits further investigation of LAM9C1 and C2 subpopulations using specific markers of RD^Rio^ strains [59,60].

**Table 3:** Allele copy number of MIRU-VNTR markers in LAM9C1 and LAM9C2 *M*. *tuberculosis* isolates.

LAM9 sublineages	Tandem repeat copy number	Number of Patients strains by MIRU-VNTR locus											
		2	4	10	16	20	23	24	26	27	31	39	40
**LAM9C1**	ND	0	3	0	0	0	0	0	0	0	1	1	0
	0	0	0	0	0	0	0	0	0	0	0	0	**119**
	1	**213**	2	2	0	7	0	226	1	2	4	1	1
	2	12	216	2	18	218	5		6	0	**172**	222	22
	3	1	5	21	**207**	1	1		29	217	49	2	1
	4			189	1		1		22	4	0		18
	5			12			7		164	3	0		19
	6			0			192		4				39
	7						18		0				6
	8						2		0				0
	9												1
**LAM9C2**	ND	0	0	0	0	1	1	0	0	0	0	0	1
	0	0	0	1	2	1	1	0	0	0	0	0	1
	1	1	1	0	33	18	0	208	0	2	0	7	**179**
	2	**200**	202	14	**123**	188	2		7	35	24	197	1
	3	7	5	42	47	0	13		25	165	**176**	4	23
	4			135	3		2		40	4	5		3
	5			15			24		114	2	3		0
	6			1			156		18				0
	7						8		2				0
	8						1		2				0
	9												0

ND: Not done

## Conclusions

By analyzing “classical” genotyping results extracted from an international database, we were able for the first time to reveal structuration of LAM9 sublineage into two distinct subpopulations LAM9C1 and LAM9C2 in the Americas. These clusters are characterized by contrasted geographical distribution, allelic richness, expansion ratios, and expansion dating estimates. Considering the combination of these characteristics, one may hypothesize that two distinct sublineages exist within the LAM9. Further studies based on WGS of LAM strains will allow one to have the needed resolution to decipher the biogeographical structure and evolutionary history of this successful family.

## Supporting Information

S1 Fig Geographic distribution of MTB lineages in various countries of Americas (when n>88). Country codes are shown as ISO 3166–1 alpha-3 code.
(TIF)

S2 Fig Geographic distribution of T sublineages in various countries of Americas (when n>31). Country codes are shown as ISO 3166–1 alpha-3 code.
(TIF)

S3 Fig Geographic distribution of H sublineages in various countries of Americas (when n>32). Country codes are shown as ISO 3166–1 alpha-3 code.
(TIF)

S4 Fig Number of subpopulation among LAM9 sublineage by calculation of delta K using the Evanno method. The maximum value is observed at K = 2.
(TIFF)

S5 Fig Minimum Spanning Tree illustrating evolutionary relationship between (A) T lineage and (B) H lineage isolates. The analysis is based on combination of spoligotypes and 12-loci MIRU-VNTR markers; the complexity of the lines denotes the number of allele/spacer changes between two patterns; the size of the circle is proportional to the total number of isolates sharing same pattern.
(TIF)

S1 Table Description of predominant SITs in this study. Only >3% of a given SIT as compared to their number in each lineage are presented.
(XLSX)

S2 Table Allelic richness ± standard deviation (SD) of main MTB lineages and sublineages. Allelic richness is evaluated for 12-loci MITU-VNTRs using a rarefaction procedure (when n>27 per lineage).
(XLSX)
