# Supplementary figures and images for: Genetic Structuration, Demography and Evolutionary History of Mycobacterium tuberculosis LAM9 Sublineage in the Americas as Two Distinct Subpopulations Revealed by Bayesian Analyses
Source: PLoS One. 2015 Oct 30;10(10):e0140911. doi: 10.1371/journal.pone.0140911 (PMC4627653; doi:10.1371/journal.pone.0140911)

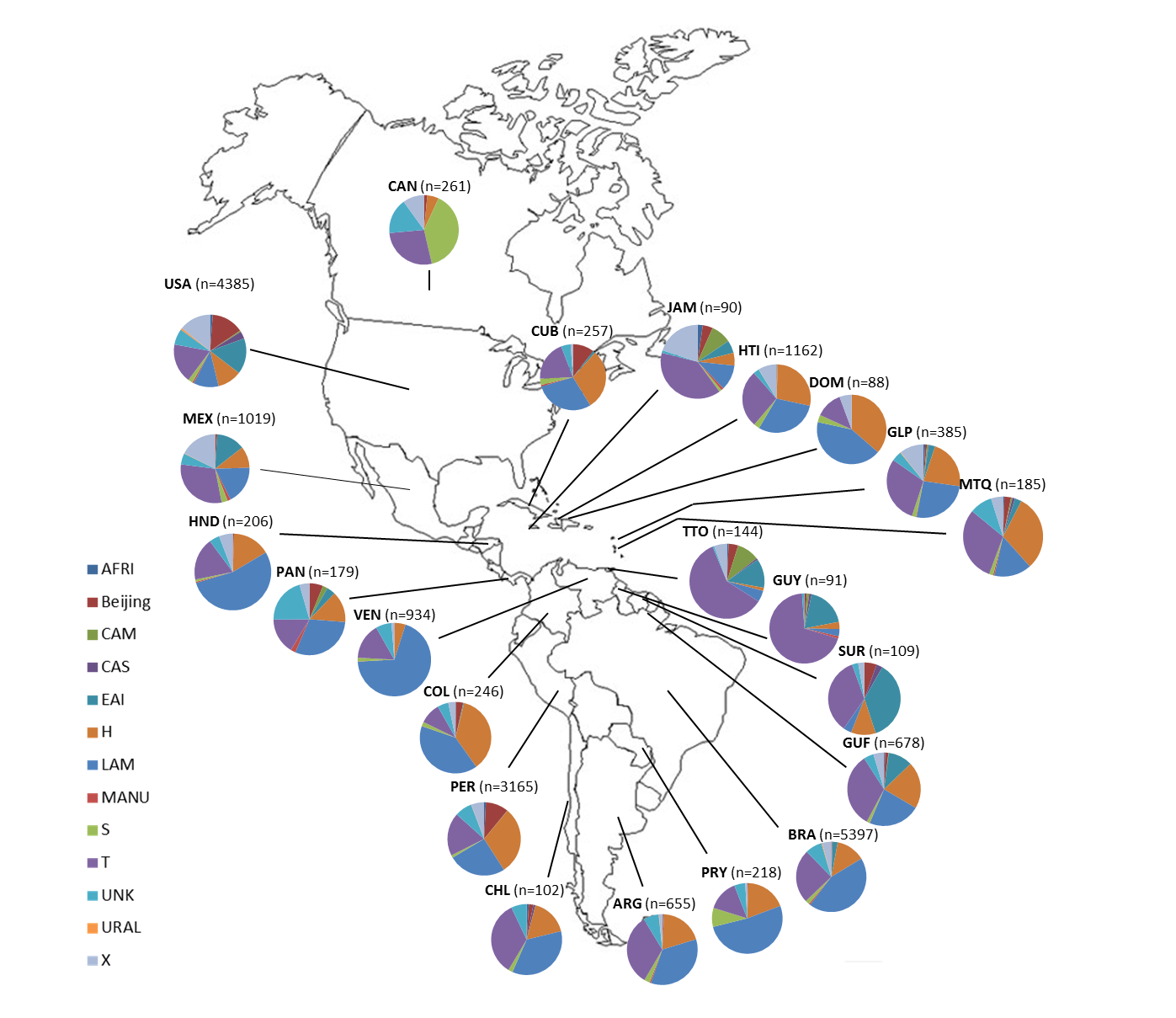

Supplement: S1 Fig — Country codes are shown as ISO 3166–1 alpha-3 code. (TIF) [file pone.0140911.s001.tif]

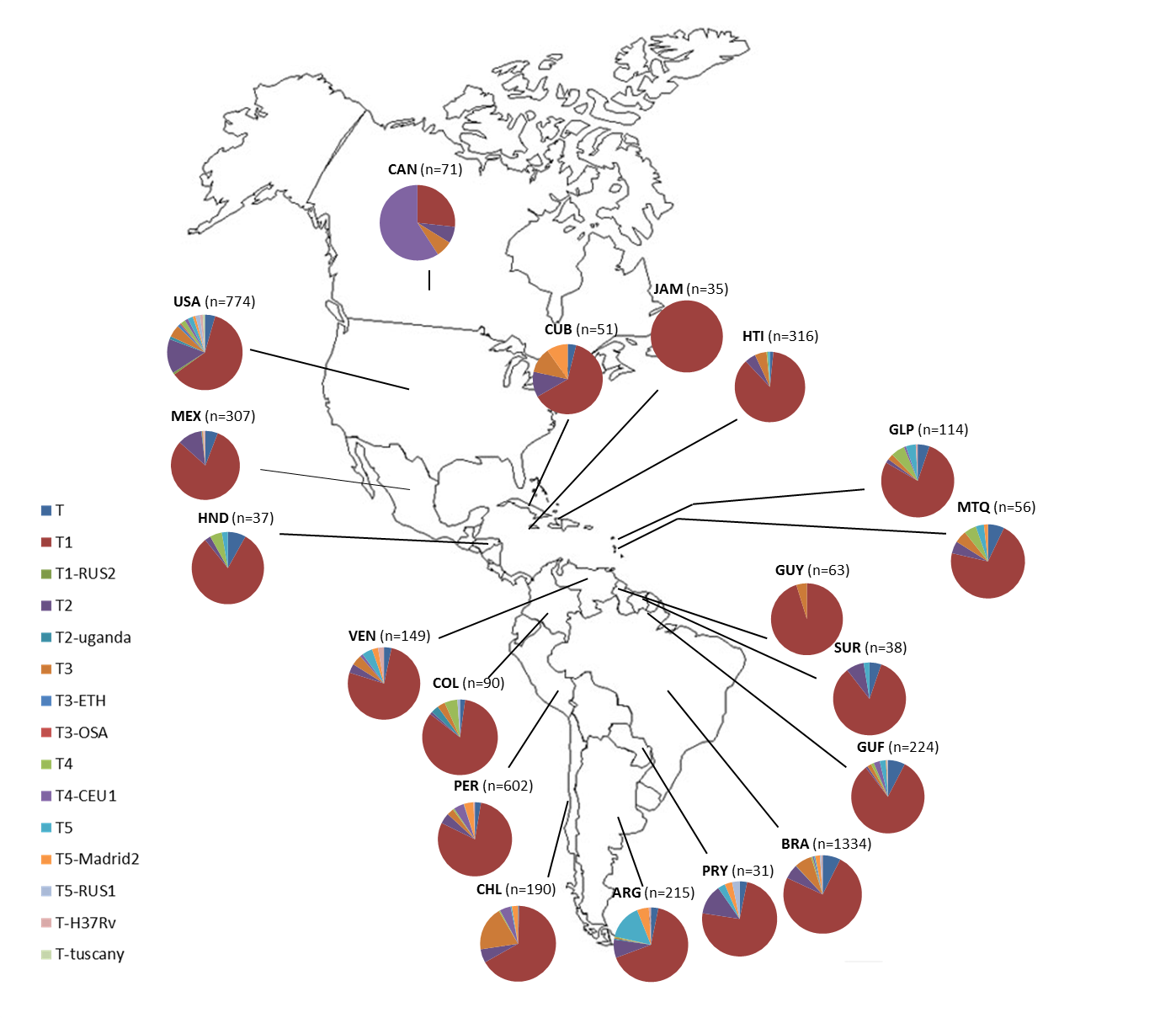

Supplement: S2 Fig — Country codes are shown as ISO 3166–1 alpha-3 code. (TIF) [file pone.0140911.s002.tif]

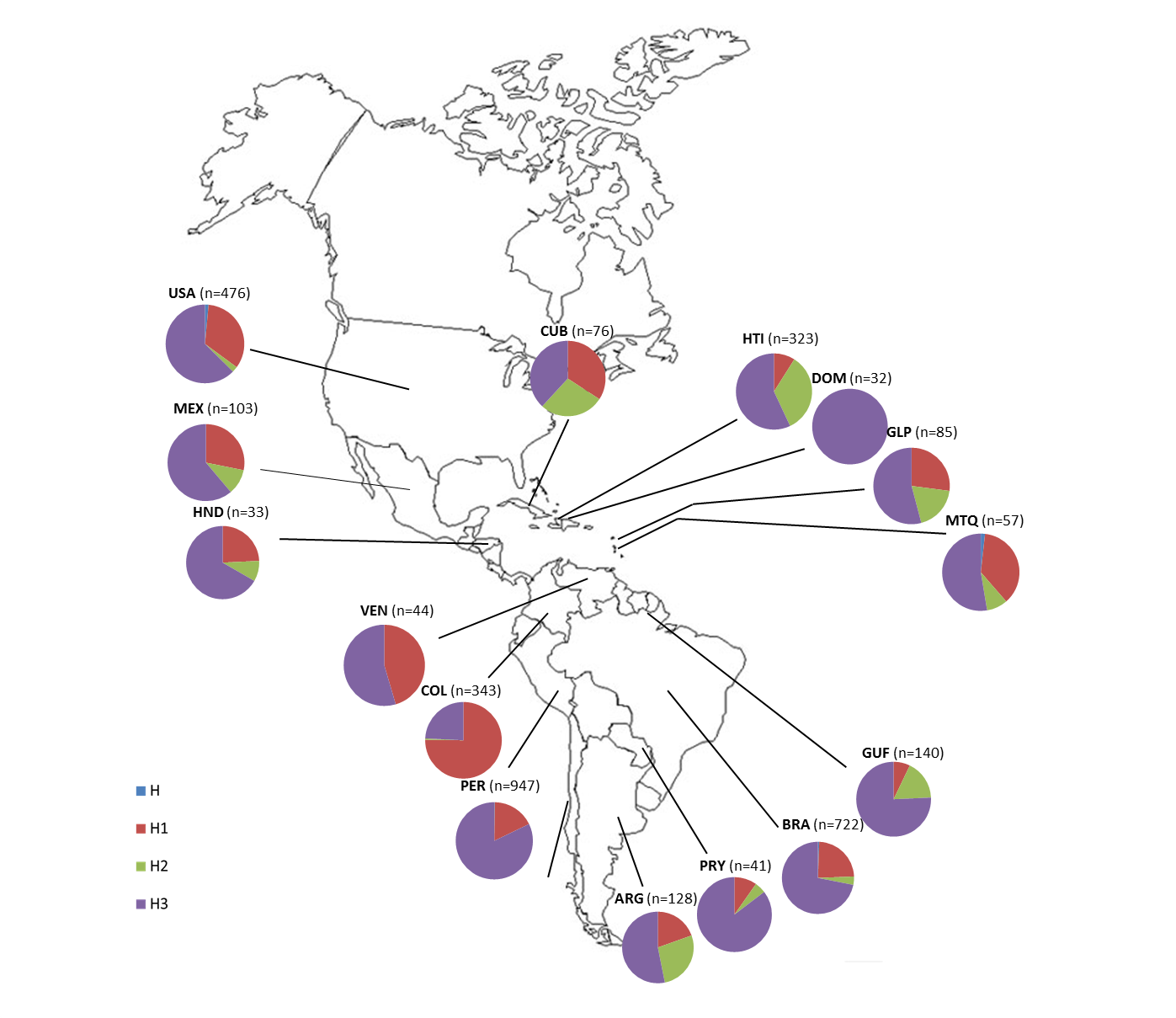

Supplement: S3 Fig — Country codes are shown as ISO 3166–1 alpha-3 code. (TIF) [file pone.0140911.s003.tif]

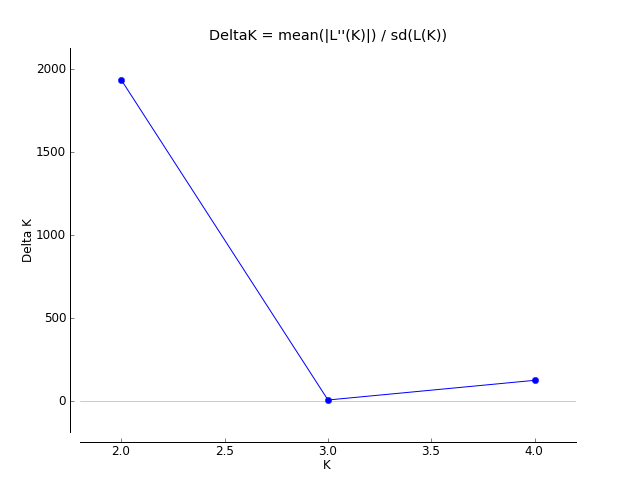

Supplement: S4 Fig — The maximum value is observed at K = 2. (TIFF) [file pone.0140911.s004.tiff]

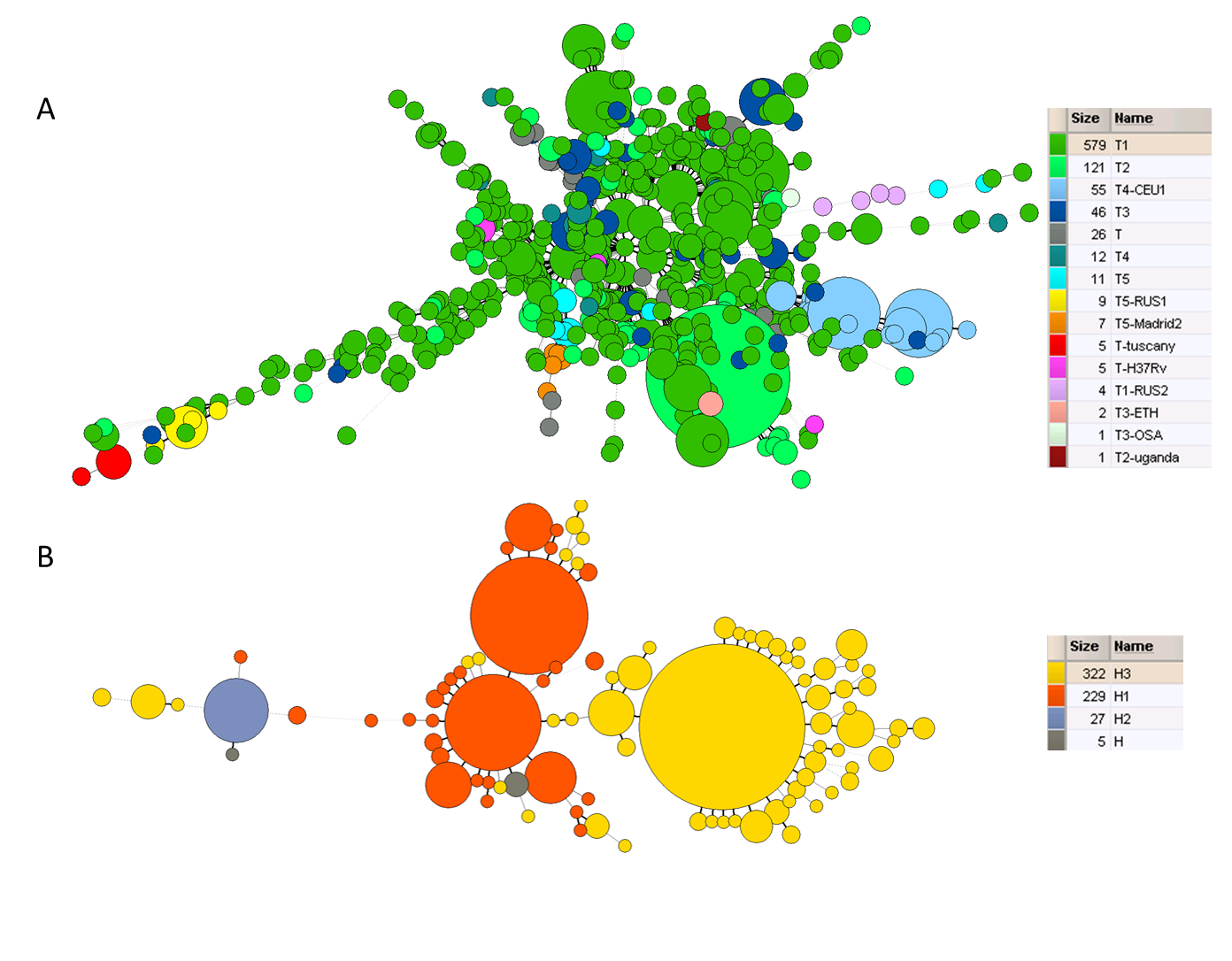

Supplement: S5 Fig — The analysis is based on combination of spoligotypes and 12-loci MIRU-VNTR markers; the complexity of the lines denotes the number of allele/spacer changes between two patterns; the size of the circle is proportional to the total number of isolates sharing same pattern. (TIF) [file pone.0140911.s005.tif]
